# Healthy Ecosystems Are a Prerequisite for Human Health—A Call for Action in the Era of Climate Change with a Focus on Russia

**DOI:** 10.3390/ijerph17228453

**Published:** 2020-11-15

**Authors:** Dmitry Orlov, Marija Menshakova, Tomas Thierfelder, Yulia Zaika, Sepp Böhme, Birgitta Evengard, Natalia Pshenichnaya

**Affiliations:** 1 Faculty of Geography, Lomonosov Moscow State University, 119991 Moscow, Russia; 2 Department of Natural Sciences, Murmansk Arctic State University, 183038 Murmansk, Russia; dendrobium@yandex.ru; 3 Department of Energy & Technology, Swedish University of Agricultural Sciences, 75007 Uppsala, Sweden; tomas.thierfelder@slu.se (T.T.); sepp.bohme@slu.se (S.B.); 4 Federal Research Centre «Kola Science Centre of the Russian Academy of Sciences», 184209 Apatity, Russia; yzaika@inbox.ru; 5 Department of Clinical Microbiology, Umeå University, 90187 Umeå, Sweden; birgitta.evengard@umu.se; 6 Central Research Institute of Epidemiology, 111123 Moscow, Russia; natalia-pshenichnaya@yandex.ru

**Keywords:** zoonotic diseases, climate change, Arctic, Russia, health care system

## Abstract

Throughout history, humans have experienced epidemics. The balance of living in nature encircled by microorganisms is delicate. More than 70% of today’s emerging infections are zoonotic, i.e., those in which microorganisms transmitted from animals infect humans. Species are on the move at speeds never previously recorded, among ongoing climate change which is especially rapid at high latitudes. This calls for intensified international surveillance of Northern infectious diseases. Russia holds the largest area of thawing permafrost among Northern nations, a process which threatens to rapidly disrupt the balance of nature. In this paper, we provide details regarding Russian health infrastructure in order to take the first steps toward a collaborative international survey of Northern infections and international harmonization of the procured data.

## 1. Introduction

The primary perspectives on climate, ecosystem functionality and transitioning landscapes began to form at the turn of the eighteenth and nineteenth centuries. Alexander von Humboldt was the first scientist to talk about harmful human-induced climate change. This German investigator born in 1769 was the founder of “ecosystem sciences”. During his Russian expedition in 1829, von Humboldt listed three ways that humans were affecting the climate at that time: through ruthless irrigation, deforestation and the use of steam and gas for industrial purposes [1]. Svante Arrhenius (1859–1927) further developed von Humboldt’s ideas on the possible emergence of an Anthropocene era as a result of the industrial revolution which started in 1769 [2]. However, the Swedish scientist thought that this would take several thousand years. Today, we know that it is happening right now and at the fastest speed in the Arctic. The world has a lot to learn from what is happening up north.

## 2. Climate Change in the Arctic

According to the Intergovernmental Panel on Climate Change (IPCC) [3,4], warming in the Arctic is happening twice as fast as in the rest of the world. Regarding hot extremes, the strongest warming is expected to occur at high latitudes in the cold season (with increases of up to 4.5 °C with 1.5 °C of global warming, i.e., a factor of three) [4]. The effects of climate warming have been clearly felt in northern communities of the Arctic, where the environment, people and animals have been affected [5,6,7,8,9]. This process also has an important impact on climate change in other parts of the world [4].

Since 2007, some ancient sites of permafrost in the Arctic have warmed by more than 0.5 °C [10]. The season of stable snow cover has become shorter. These changes affect local people and ecosystems in a significant fashion [11]. The global surface temperature has warmed by 0.85 °C on average since 1880 [3]. A higher rate of warming in the Arctic is caused by feedback loops that are unique to the Far North. For example, when ice melts in the Arctic summer, seawater absorbs more solar heat, which leads to faster ice melting.

Permafrost is ground that continuously remains frozen for two or more years [10]. Almost a quarter of the Northern Hemisphere is underlain by permafrost [12], and the largest area is in Russia. The current area of permafrost in the Northern Hemisphere is approximately 15 million km^2^. This is predicted to decrease to 12 million km^2^ by 2040, followed by a rapid decrease to between 5 and 8 million km^2^ by 2080 [10]. Melting permafrost leads to coastal erosion and unstable mountain slopes, and seriously threatens human settlements, infrastructure and objects of cultural heritage [10,13].

Frozen ground across the globe holds an estimated 1500 billion tons of carbon—twice as much as is contained in the atmosphere [14]—and half the world’s total soil carbon [10]. This reservoir of carbon remains stable as long as the ground is frozen. However, as the permafrost thaws, the amount of carbon emitted into the atmosphere will increase [14]. This will lead to an increase in forest and tundra fires and a loss of natural habitats. Such changes will force species to move to new territories, carrying with them zoonotic infections [15].

This thawing trend appears to be irreversible. Under an IPCC high greenhouse gas emissions scenario, stable permafrost will most likely remain only along the Arctic coasts of Russia and Canada and in the uplands of East Siberia [10]. Most climate models predict that, at the current rate of carbon emission, there will be no summer ice in the Arctic as early as the 2030s [10,16]. Warming and desalination due to the melting of glaciers in the Arctic Ocean will reduce the formation of cold deep-strata water, which could weaken the Gulf Stream [17]. This may affect global weather systems. The frequency of strong hurricanes, forest fires and heat waves is currently increasing in the Northern Hemisphere [18,19]. People living in the Arctic are already experiencing these changes. Adaptation to changing climatic conditions has already become a part of their daily life [11,20]. Indigenous peoples from Arctic regions, such as the Inuit in Greenland, the Sami in Sápmi and the Nenets, Evenki, Chukchi and others in Russian Siberia, have all developed adaptations to the changing environment. [21].

Rapid reaction to climate change usually demands long-term data collectionin order to assess pre- and post- climate change trends at the level of ecosystems and species [22]. Fortunately, research and monitoring stations have been operating around the Arctic for some time. These stations were organized under the EU project called INTERACT (International Network for Terrestrial Research and Monitoring in the Arctic). INTERACT aims for a modern and geographically comprehensive terrestrial research infrastructure in the Arctic and adjoining regions to identify environmental changes, facilitate understanding and prediction of future changes and inform decision makers about societally-relevant impacts (see http://www.interact-eu.net/#o=about).

## 3. Species on the Move

Large global species redistributions associated with climate change have been taking place since the last glacial maximum. The geographical ranges of species naturally fluctuate in space and time, but recent climate changes could cause massive movements of animal populations to new territories. If cold-loving species cannot adapt to warming local conditions, they will seek new habitats closer to the poles, higher in the mountains or deeper in the oceans. [23]. On average, terrestrial taxa move poleward by 17 km per decade [6] and marine taxa by 72 km per decade [7,24], as meta-analyses have shown. Just as terrestrial species on mountainsides are moving upslope to escape warming lowlands [25], some fish species are now being driven deeper as the sea surface is warming [26].

## 4. Zoonoses: Diseases Common to Animals and Humans

One Health (an initiative committed to designing and implementing programs, policies, legislation and research in which multiple sectors communicate and work together to achieve better public health outcomes; https://www.onehealthinitiative.com) takes a holistic approach to health risks and their mitigation among humans, animals and in the environment. The One Health concept emphasizes that human health and welfare are dependent on ecosystem health.

Pandemics occur as a result of the emergence of new diseases to which people do not have immunity. Zoonoses transmitted from animals to humans play an important role [27,28]. More than two thirds of current human infections and many of the emerging infections in northern regions are zoonotic [29].

Zoonoses may be caused by bacteria, viruses, parasites, fungi or prions (proteins linked to several fatal neurodegenerative diseases). They have different means of transmission, including through direct contact between animals and humans, hematophagous arthropods, intake of food and water contaminated with parasites and through the air [30]. The Spanish flu of 1918, caused by the Influenza A virus found naturally in wild aquatic birds, claimed between 30 and 50 million lives [31] and is perhaps the best-known and deadliest example of a zoonosis. Other examples include the rabies virus and recent emerging diseases such as the Ebola and Zika viruses, as well as the ongoing pandemic caused by the SARS-CoV-2 virus [32].

The number of nosoforms observed in Arctic species has increased in recent years. These include avian cholera outbreaks in marine birds in Alaska and the Canadian Arctic Archipelago, as well as mortalities within walrus and seal populations in the U.S. Arctic [33]. Thawing permafrost can potentially release anthrax spores, as was seen in 2016 in the Yamal Peninsula in the Russian Arctic. This outbreak was the largest in decades and resulted in the death of a 12-year-old boy, the hospitalization of around 100 people, laboratory confirmed diagnoses of anthrax in 36 patients and the deaths of 2300 reindeer [34]. There is, furthermore, a risk of the appearance of ancient microorganisms unknown to science as a result of the thawing of permafrost [35,36,37]. Migratory birds also have the potential to transmit ticks across long distances along with antibiotic resistant genes.

The spread of zoonotic diseases can take a major economic toll on many industries, including those in the manufacturing, agricultural, travel and hospitality sectors. Furthermore, the peace and economic stability of communities is both directly and indirectly connected to disease outbreaks [38].

The connection between human and animal microbiology was originally recognized by Virchow and Osler in the nineteenth century [39], and the connection of diseases with ecosystems (natural focal disease theory) was posited by Pavlovsky in the first half of the twentieth century [40]. However, it is only recently that this relationship has been emphasized through the One Health initiative, which promotes equitable collaboration between practitioners and researchers in human and comparative medicine and in wildlife, domestic animal and human observation programs. Despite the fact that the term “One Health” is quite new, this concept has already gained recognition both nationally and globally [41].

According to the WHO, the majority of zoonoses arise in natural ecosystems. Due to growing human involvement in natural landscapes, natural foci of infections, which have undoubtedly existed for a long time, have become more active. This problem is especially pressing because of the extensive incorporation of previously untouched territories in agriculture and recreational activities, as well as increased rates of tourism and migration [42]. Zoonoses can be classified according to the ecosystem in which they circulate. These diseases are either synanthropic zoonoses, denoting an urban (domestic) cycle in which the sources of infection are domestic and synanthropic animals (e.g., cat scratch disease, zoonotic ringworm and urban rabies), or exoanthropic zoonoses, denoting a sylvatic (wild and feral) cycle in natural foci outside human habitats (e.g., Lyme disease, arboviroses, tularemia and wildlife rabies). However, some zoonotic diseases can circulate in both natural and urban cycles (e.g., Chagas disease and yellow fever). Many zoonoses can be attributed to anthropogenic changes. The loss of wildlife habitats to development and the consumption of bushmeat, necessitated by poverty or resulting from cultural preference, increase opportunities for cross-species jumps. Climate warming may also increase the geographic range of arthropod vectors, such as ticks and mosquitoes, which serve as vectors and reservoirs for infectious agents [32].

Zoonotic disease management requires the collaboration of different types of disease-control and health specialists, since zoonotic disease agents can exist in humans, animals and the environment. Disease control should include host-control (mammals, birds, fishes) and vector-control (ticks, fleas, mosquitoes) programs; environmental cleanup or protection may also be required to control disease agents that remain viable on different surfaces, in water or in the soil [32].

New infectious diseases seem to be inevitable and previously rare ones are increasingly seen in unexpected places due to global warming, deforestation, human intrusion into previously underexplored or never-explored sites, world travel and the increasing threat of biological terrorism or warfare. Increased human contact with wild and domestic animals, population growth and urbanization and a lack of necessary sanitation can increase the incidence and severity of zoonotic diseases [43].

In 2019, a Nordforsk-funded Nordic Center of Excellence, Clinf (clinf.org) arranged a seminar with Russian colleagues at the Lomonosov Moscow State University Faculty of Geography focusing on the impacts of the transmission of infectious diseases in the North. It was decided that the discussions should be published as a paper with a focus on Russian research infrastructures in order to facilitate further collaborations.

## 5. International Collaboration Needed for Surveillance

A dataset produced by CLINF showing the variability of certain diseases by country and over time is presented in Table 1 [44].

As is clearly shown here, despite the obvious need for the harmonization of surveillance data, there is still a long way to go before international comparisons can be quickly and efficiently made in order to project and predict, and even prevent, outbreaks. Even in a part of the world where infrastructures are quite similar, as in the Nordic countries, surveillance data are far from harmonized. With the largest tundra in the world (in Russia) thawing, collaboration with Russian colleagues and harmonization with Russian health databases and infrastructures are essential. As these aspects of Russian health care are still quite unknown to many, here, we provide details of the pertinent infrastructures and suggest how collaboration could be approached. There are no data on gender and age among the official open data in Russia, but these can be found through the local (regional) departments (Appendix A).

## 6. Russia in Detail

**Figure 1:**
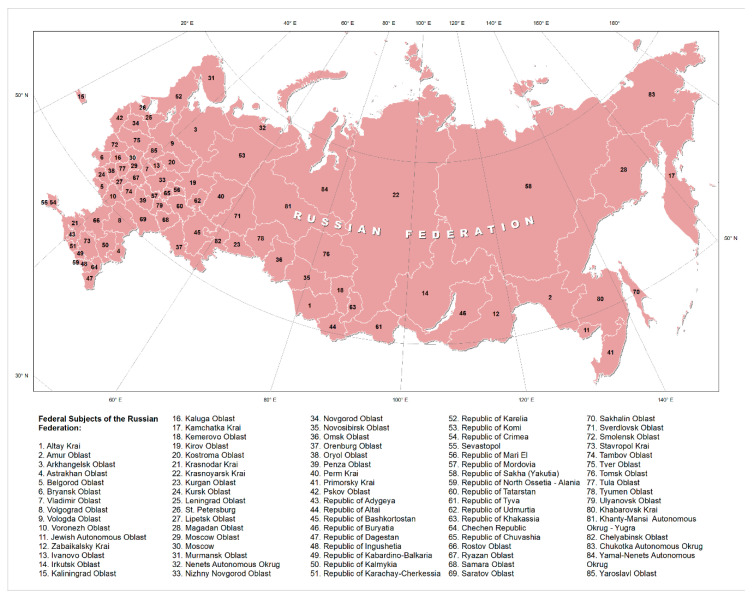
Administrative divisions of Russia.

Russia has the largest territory (17,125,191 sq km) in the world and covers more than one eighth of the habitable part of the Earth. It consists of 46 oblasts (provinces); 22 republics; 9 krais (territories); 4 autonomous okrugs (districts); 1 autonomous oblast; and 3 federal cities (Figure 1).

**Figure 2:**
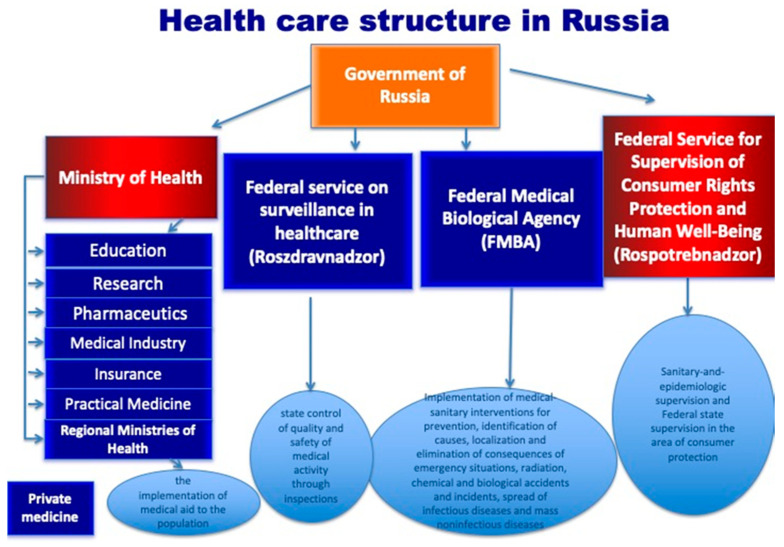
Health care structure in Russia.

The health care system in Russia consists of two main institutions, the Ministry of Health (MoH) and the Federal Service for Supervision on Consumer Rights Protection and Human Wellbeing (Rospotrebnadzor), as well as two institutions which were separated from the MoH at the beginning of 2020, the Federal Service for Surveillance in Healthcare (Roszdravnadzor) and the Federal Medical Biological Agency (FMBA). The MoH is responsible for the implementation of medical aid for the population, medical education, research, the pharmaceutical and medical industries and control of private medicine. Rospotrebnadzor is responsible for sanitary and epidemiological supervision and federal state supervision in the area of consumer protection (Figure 2). Roszdravnadzor is responsible for state control of the quality and safety of medical activity through inspections and through the FMBA, which is, in turn, responsible for the implementation of medical-sanitary interventions for the management of emergency situations (including prevention, identification of causes, localization and elimination of consequences); radiation, chemical and biological accidents and incidents; and the spread of infectious diseases and mass noninfectious diseases.

**Figure 3:**
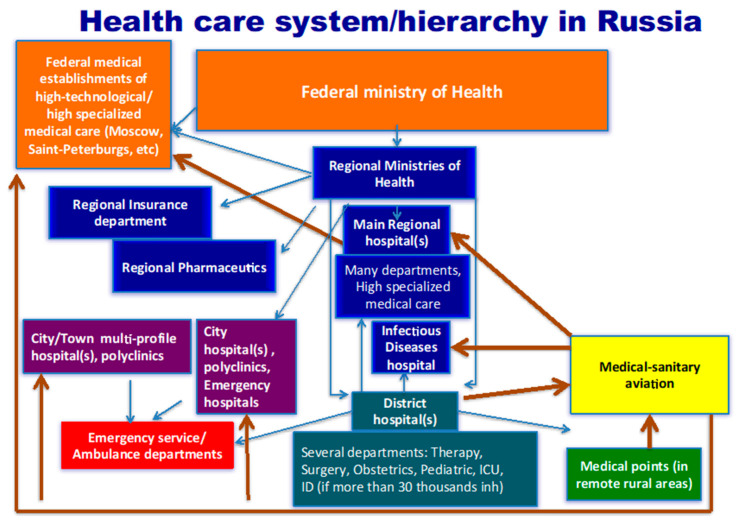
Health care system/hierarchy in Russia.

The health care system of the MoH consists of the federal level (level IV)—the Federal MoH and the federal-level institutions directly subordinate to it that provide high-tech assistance to the population; the Regional level (level III)—the regional MoH, main regional hospitals with many departments which provide highly specialized medical care, regional insurance departments and regional pharmaceutics; the town/district level (level II)—town/district hospitals with several departments (emergency room, therapy, surgery, obstetrics, pediatrics, infectious diseases, ICU, neurology, etc.); and the primary care level (level I)—medical points (in remote rural areas), medical offices and policlinics. Emergency/ambulance services and medical sanitary aviation function across all levels of the medical system (Figure 3).

Rospotrebnadzor investigates outbreaks and cases of infectious diseases at all levels of medical care, inspects laboratory diagnostics of infectious diseases(especially dangerous infections), conducts routine monitoring of the epidemiological situation(as well as of natural foci of zoonotic infections) and implements the assessment and monitoring of the quality of food, air and water.

Any doctor or nurse who suspects the presence of any infectious disease in a patient at any level of medical care, in any medical establishment, must send an initial emergency notification to the epidemiological department of the territorial department of Rospotrebnadzor. The patient will be hospitalized or transferred to an infectious disease unit or hospital if isolation is necessary or if the severity of the patient’s condition requires it. The patient’s samples of biological fluids will be sent for laboratory testing. After the results of the laboratory tests are produced, a second emergency notification with information about confirming, excluding or changing the diagnosis is sent to Rospotrebnadzor. In parallel with treatment in medical facilities by specialists from the MoH, Rospotrebnadzor conducts investigations into cases and outbreaks of infectious diseases. Specialists from the MoH may also be involved in this process, for example, by providing emergency vaccination or conducting examinations of contact persons, etc. Rospotrebnadzor provides all summary data about cases of infectious diseases to the MoH on a monthly basis. Rospotrebnadzor also provides regular reports on infectious diseases (there are more than 100 nosologies and postvaccinal complications on its regional and federal websites).

The Program for Monitoring Emerging Diseases (ProMED-mail) was organized in 1994 as an outbreak reporting system based on web technologies. In 1999, ProMED-mail started to operate as an official program of the International Society for Infectious Diseases. ProMED-mail is now an electronic outbreak reporting system for the monitoring of emerging infectious diseases globally. It acquires information from all open official and unofficial sources and works 24 h a day and seven days a week. ProMED-mail often announces outbreaks through public access systems before official notifications and makes information on them accessible to the public.

The ProMED-mail Russian language service started in 2004. The Russian language network covers Russia and the former USSR countries. The ProMED-mail RUS team provide between one and three posts every day about disease outbreaks or unusual events relating to infectious diseases in Russia and the countries of the former Soviet Union, which are distributed for free to more than 1000 subscribers. Subscription to the service is also free. Rospotrebnadzor frequently uses information posted in ProMED-mail about unusual events relating to infectious diseases abroad in its public reports. ProMED-mail RUS provides valuable contributions to the official epidemiological surveillance system in Russia for use in practical work.

## 7. Conclusions

Although it is difficult to encompass, all local, regional and global decision-making regarding climate change countermeasures and planning must consider and include the resulting biological/ecological chain reaction.

Epidemics have plagued humans throughout history. The balance of living in nature surrounded by microorganisms is delicate. Diseases can emerge when this balance is shifted by the various causes that occur when humans come into closer contact with previously uninhabited areas. Climate warming, which is currently indisputable, can accelerate this process. International surveillance of infectious diseases needs to be improved. However, as shown in this paper, despite the obvious need for the harmonization of surveillance data, there is still a long way to go before international comparisons can be quickly and efficiently made in order to project, predict and even prevent outbreaks.

We believe that the first step for data harmonization should be an international conference with the participation of the Arctic states’ health authorities. We understand that it is difficult to hold such a conference at the global level (WHO). To begin with, it may be possible to hold such a conference at the level of Europe and Russia (the European Centre for Disease Prevention and Control (ECDC) and Rospotrebnadzor).

As we have shown in this study, well-functioning international collaboration in surveillance and data collection is needed. We also point to the need for the involvement of local communities in such efforts. We have created a database that can be used by international experts from a variety of disciplines, as addressing climate-sensitive infections (CSIs) requires a multidisciplinary approach. There are no administrative borders for microorganisms and species, and in order to keep infectious diseases under control, we need broad and trustful international collaboration and harmonization of collected data.

## Figures and Tables

**Table 1:** Additional information concerning gender and age per nation and disease.

Nation	BOR	BRU	CRY	LEP	PUU	QFE	TBE	TUL
Finland	1995–2016	1995–2014	1995–2016	1995–2016	1995–2016	1998–2016	1995–2016	1995–2016
Greenland	n/a	n/a	n/a	n/a	n/a	2007–2007 *	n/a	n/a
Iceland	n/a	n/a	-	n/a	n/a	n/a	n/a	n/a
Norway	1990–2016	2004–2016	2012–2016	n/a	1991–2016	n/a	1998–2016	1985–2016
Russia	-	-	n/a	-	-	-	-	-
Sweden	-	-	2004–2016	-	1985–2016	-	1978–2016	1969–2016

n/a (not applicable) indicates that the disease has not been reported. (-) indicates a lack of supplementary information despite reported diseases. Ixodid tick-borne borrelioses (BOR); brucellosis (BRU); cryptosporidiosis (CRY); leptospiroses (LEP); hemorrhagic fever with renal syndrome/Puumala virus infection (PUU); Q fever (QFE); tick-borne encephalitis (TBE); tularemia (TUL). * = A single case of QFE reported in Greenland 2007.

## Appendix A

### The Russian Statistical System

The general Russian statistical system has a structure which is organized through a bottom-up framework. Socioeconomic, well-being and health (e.g., the number of municipal hospitals, patients, health care funding, etc.) indicators are incorporated by the Federal Service for State Statistics (www.rosstat.gov.ru) in the form of reports for the whole territory of the Russian Federation and regionally at local statistical centers. More specific indicators on infectious diseases can be found through the state services which monitor the epidemiological situation in Russia. Rospotrebnadzor monitors cases of infectious diseases among the human population while Rosselkhoznadzor (Federal Service for Veterinary and Phytosanitary Surveillance) detects animal cases.

However, the bottom-up framework is the basis for all informational systems and services. While generalized state reports contain information from the wider geographical perspective, regional divisions of services provide more detailed and specific information on the epidemiological situation within the defined regions. Such information is collected in the form of annual reports which are structured in an electronic (Excel spreadsheet) format with tables, graphs and diagrams. The current long-term database format which exists in European countries has been applied in the Russian system only during last 20 years, while the rest of the information for previous years is stored on paper and can be obtained only locally and processed manually. The digitalized information is open access and can be found online. If a particular annual report is not available, it is necessary to officially apply to the state services for access.

The indicators for multiple different infectious diseases are included in the reports according to the region in which cases occurred (registered clinically-approved cases). However, to make such data available in a suitable format, experts should manually extract the separate statistics from the reports into a table which can be used as part of a research method.
